# Gelled Electrolyte Containing Phosphonium Ionic Liquids for Lithium-Ion Batteries

**DOI:** 10.3390/nano8060435

**Published:** 2018-06-14

**Authors:** Mélody Leclère, Laurent Bernard, Sébastien Livi, Michel Bardet, Armel Guillermo, Lionel Picard, Jannick Duchet-Rumeau

**Affiliations:** 1 Université de Lyon, F-69003, Lyon, France, INSA Lyon, CNRS, UMR 5223, Ingénierie des Matériaux Polymères, F-69621 Villeurbanne, France; melody.leclere@insa-lyon.fr (M.L.); sebastien.livi@insa-lyon.fr (S.L.); 2 Université Grenoble Alpes, CEA-Grenoble, LITEN/DEHT/SCGE/LGI, 17, F-38000 Grenoble, France; Laurent.BERNARD3@cea.fr (L.B.); armel.guillermo@free.fr (A.G.); lionel.picard@cea.fr (L.P.); 3 Université Grenoble Alpes, CEA-Grenoble, INAC/SCIB/LRM, F-38000 Grenoble, France; michel.bardet@cea.fr

**Keywords:** ionic liquids, thermosets, Lithium salts, electrolytes

## Abstract

In this work, new gelled electrolytes were prepared based on a mixture containing phosphonium ionic liquid (IL) composed of trihexyl(tetradecyl)phosphonium cation combined with bis(trifluoromethane)sulfonimide [TFSI] counter anions and lithium salt, confined in a host network made from an epoxy prepolymer and amine hardener. We have demonstrated that the addition of electrolyte plays a key role on the kinetics of polymerization but also on the final properties of epoxy networks, especially thermal, thermo-mechanical, transport, and electrochemical properties. Thus, polymer electrolytes with excellent thermal stability (>300 °C) combined with good thermo-mechanical properties have been prepared. In addition, an ionic conductivity of 0.13 Ms·cm^−1^ at 100 °C was reached. Its electrochemical stability was 3.95 V vs. Li^0^/Li^+^ and the assembled cell consisting in Li|LiFePO_4_ exhibited stable cycle properties even after 30 cycles. These results highlight a promising gelled electrolyte for future lithium ion batteries.

## 1. Introduction

These last years, the development of storage energy system such as lithium ion batteries has witnessed a real boom in academic and industrial research [1,2]. In fact, rechargeable batteries with high energy/power density, cycling stability, and safety were developed [3,4]. However, many challenges must be overcome, such as the development of safe electrolytes [5]. In this case, gelled electrolytes represent a promising way to prevent leakage of liquid electrolyte when the electrolytes are used in electrochemical devices. Indeed, gelled electrolytes confine the liquid electrolyte in a polymer matrix, which plays the role of host. Thus, many works were reported and were investigated from the confinement of a conventional electrolyte (organic solvent with lithium salt) and extended to the use of ionic liquids (ILs) as a solvent [6,7]. Recently, ionic liquids (ILs) have appeared as promising candidates to replace flammable organic solvents thanks to their excellent thermal and chemical stability, their good ionic conductivity, their low vapor pressure, and their endless cation/anion combinations [8,9,10,11]. Moreover, some ILs have presented a high compatibility with linear polymer [12,13,14], such as polyethylene oxide (PEO), polyvinylidene fluoride-*co*-hexafluoropropylene (PVDF-HFP), poly(ionic liquids) PILs (polydiallylmethylammonium), and thermosets such as epoxy networks [15,16,17,18,19]. The use of a crosslinked polymer as a host polymer is an interesting route to develop polymer electrolytes with good mechanical performances despite a large quantity of introduced ionic liquids. Moreover, the main advantage of these gelled electrolytes is that no solvent is required during their processing. For example, Stepniak et al. have prepared a gelled electrolyte based on *N*-methyl-*N*-propylpiperidinium [MPPip] trifluroromethanesulfonimide [TFSI] obtained by photopolymerization of bisphenol A diacryalate [18]. These authors have highlighted the significant mechanical strength of the electrolyte even with 80 wt % of confined electrolyte and an ionic conductivity of 0.63 mS·cm^−1^. In addition, a good electrochemical performance in Li|LFP battery at 25 °C with moderate rate C/10 getting a specific capacity superior to 150 m Ah·g^−1^ was demonstrated. Other authors such as Sotta et al. have developed a gelled electrolyte based on epoxy networks (DGEBA/polyetheramine) containing 1-Butyl-3-methylimidazolium bis(trifluoromethyl-sulfonyl)imide [BMIM] [TFSI] and have obtained excellent transport properties with an ionic conductivity of approximately 0.4 mS·cm^−1^ at 20 °C [20]. However, the electrochemical stability window of imidazolium-based ILs being limited to 1.5–5 V vs. Li+/Li^0^ prevents the development of high-voltage devices.

More recently, the use of phosphonium ionic liquids as an electrolyte solvent for the energy storage system has also been investigated [21,22,23,24]. MacFarlane et al. have shown the compatibility of tributylmethylphosphonium [P1444] bis(fluorosulfonyl)imide [FSI] with metallic lithium, getting a good cycling performance at 30 °C with 160 m Ah·g^−1^ for 50 cycles at 0.03 C. But these phosphonium ionic liquids may play a significant role in the curing process of epoxy networks as well as on the final properties of the resulting thermosets [25,26,27]. Livi et al. have shown that the chemical nature of anion has a clear impact on the polymerization of epoxy networks. Indeed, counter anions such as dicyanamide [DCA] or trimethylphosphinate [TMP] act as initiators of the anionic polymerization of the epoxy prepolymer which is due to their basic nature. For these different reasons, the design of electrolyte with good transport properties and its effect on the polymerization of epoxy-amine networks are required in this study.

The main objectives of this work were to investigate the electrochemical transport properties of the gelled electrolytes based on phosphonium ILs confined in an epoxy-amine network by electrochemical impedance spectroscopy (EIS), cyclic voltammetry (CV), and galvanostatic cycling Li|LFP cell. Thus, the transport properties of phosphonium electrolytes were performed in order to determine the ionic conductivity as well as the diffusion coefficient by Nuclear Magnetic Resonance (NMR). Then, the influence of phosphonium electrolyte on the polymerization kinetics of a rubbery epoxy network was investigated. Their thermal and thermo-mechanical behaviors were also studied by dynamical mechanical analysis (DMA) and thermo-gravimetric analyses (TGA).

## 2. Materials and Methods

### 2.1. Materials and Characterization Methods

Table 1 reviews all the structure and properties of the materials used in this study. Epoxy prepolymer based on Diglycidyl ether of polypropylene glycol (PPO) was purchased from Sigma Aldrich (St. Quentin Fallavier, France). The hardener used for the curing process is Jeffamine^®^ D-2000 polyoxypropylene-diamine, and was supplied by Huntsman (Everslaan, Belgium). The ionic liquids (ILs) denoted trihexyl(tetradecyl)phosphonium bis(2,4,4-trimethyl-pentyl)phosphinate [P_66614_][TMP] and tri-hexyl(tetradecyl)phosphonium bis (trifluoromethanesulfonyl) imide [P_66614_][TFSI] were supplied by Cytec, Inc (Thorold, ON, Canada). Their structures have quaternary phosphonium cations with n-alkyl chains combined with different anions, phosphinate [TMP] and trifluoromethanesulfonimide [TFSI]. The lithium salts denoted lithium bis(2,4,4-trimethyl-pentyl)phosphinate LiTMP and lithium bis(trifluoromethanesulfonyl)imide LiTFSI were supplied by Solvionic (Toulouse, France) and Sigma Aldrich, respectively.

*Near-infrared spectroscopy* (*NIR*) was recorded by using a Bruker Spectrometer (Marne la vallée, France) equipped with a heating cell to monitor changes in adsorption in the region of 10,000 to 4000 cm^−1^ with a resolution of 4 cm^−1^ on 32 scans. A temperature control was used to monitor the curing of the sample in the same conditions. The reactive mixture was injected in a glass cell with a path-length of 8 mm when the controller reached the curing temperature.

*Dynamic mechanical thermal analysis* was performed using a Rheometrics Solid Analyser RSA II (TA instruments, New Castle, DE, USA) at 0.01% tensile strain and a frequency of 1 Hz. The heating rate was 3 °K·min^−1^. The temperature range was [−70 °C, 20 °C] in the case of epoxy/amine systems in stoichiometric ratio and [−70 °C, 70 °C] for the epoxy/amine systems in sub-stoichiometric ratio.

*Thermogravimetric analysis* (*TGA*) of Epoxy-Jeffamine/[ILs] blends were performed on a Q500 thermogravimetric analyzer (TA instruments, New Castle, DE, USA). The samples were heated from 30 to 550 °C at a rate of 20 K·min^−1^ under nitrogen flow.

**Scheme 1:**
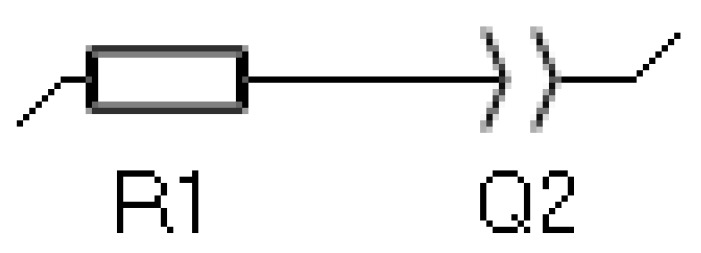
Model circuit used for the fitting of Nyquist plots.

*Ionic conductivity* was measured between aluminum electrodes by electrochemical impedance techniques using VMP 3-BioLogic (Seyssinet-Pariset, France). AC impedance measurement was measured over the frequency range from 106 Hz to 10 mHz with the potential amplitude of 50 mV. In order to investigate the temperature dependence of ionic conductivity for the electrolyte gel, the measurement was carried out on a range of temperatures between 20 and 220 °C. As the EIS spectrum did not present any semi-circle because the electrolyte was a sufficient conductor and the electrodes were blocking. Thus, the different Nyquist plots were fitted with a common model where the model circuit used is shown in the Scheme 1 as follows:

where R1 corresponding to Rel.

*Electrochemical stability windows* (*ESWs*) of the gelled electrolyte and neat polymer were evaluated by cyclic voltammetry in a linear sweep at a sweep rate of 0.05 Mv·s^−1^ at 100 °C. The measurements were performed using the VMP 3-Biologic system.

*Electrochemical stability window of gel material:* Starting from Open Circuit Voltage, the ESW was measured in coin cells in oxidation by cycling at 0.05 mV/s @ 100 °C from OCV to 4 V vs. Li^+^/Li for 20 cycles. A similar experiment was made in reduction, on other coin cells, by cycling at 0.05 mV/s @ 100 °C from OCV to 0.05 V vs. Li+/Li for 20 cycles.

The electrochemical performance of the Li|LFP cells was characterized using galvanostatic charge/discharge tests using VMP 3-Biologic system at the same potential range from 2.5 to 4.1 V at 100 °C.

The ^31^P and ^7^Li high-resolution liquid state NMR experiment were performed using an Avance DPX 400 spectrometer (Bruker, Wissembourg, France), operating at 400 MHz for ^1^H. For the scaling of ^31^P and ^7^Li chemical shifts, their 0 ppm references were set up using H_3_PO_4_ and LiCl D2O solutions respectively.

^1^H and ^7^Li Pulsed Field Gradient NMR measurements were performed with an Advance Bruker spectrometer operating at 200 MHz for ^1^H nucleus and 70.8 MHz for ^7^Li. A multinuclear self-diffusion probe was used (Bruker Diff30 probe, gradient coil equal to 0.3 T·m^−1^·A^−1^). The maximum magnetic field gradient was 12 T·m^−1^. A standard stimulated echo sequence with a diffusion time in between 10–100 ms was used [28]. The diffusion constant was obtained from the gaussian attenuation of the NMR spectrum integral versus the field gradient increase. The sample temperature controller was a Bruker BVT3000 with a+/−0.1 °C stability. Solid-state ^7^Li NMR was carried out on the same spectrometer using a 7 mm CPMAS probe, the rotors were filled with the sample to analyze. Direct ^7^Li excitation with high power proton decoupling during signal acquisition was applied.

### 2.2. Preparation of Gelled Electrolyte

The network was prepared from a mixture of amine and epoxy prepolymer with a stoichiometric ratio of 1.56. The homogenization of the mixture was carried out by magnetic stirring at 80 °C. In a first step, the epoxy-amine mixtures were precured during 6 h at 110 °C. In a second step, different contents (from 50 to 70 wt %) of phosphonium ILs were added to the precured system under mechanical stirring at 80 °C for 10 min. The solutions were degassed in an ultrasonic bath for 10 min and poured into a silicon rubber mold or coated on a PET (polyethylene terephthalate) sheet. The cure was performed at 130 °C for 6 h (to complete the polymerization).

In addition and to avoid the moisture content of the ionic liquids dramatically modifying both the ionic conductivity as well as the viscosity, all the preparation of the gelled electrolytes and the conductivity measurement have been performed in an argon-filled glove box or in coin cells assembled in an argon-filled glove box. Prior to this entry in the glove-box, the IL and the Li salt have been dried under a vacuum at 120 °C for 48 h, to remove any traces of moisture.

### 2.3. Preparation of the Cathode (LiFePO_4_) and Assembly of the Cell

The cathode was prepared by casting a slurry of LiFePO_4_ (Sigma-Aldrich) as an active material (45 wt %), filled with carbon black (5 wt %) and gelled electrolyte precured as a binder (50 wt %) in *N*,*N*-Dimethylformamide (DMF, Sigma-Aldrich) on an aluminum current collector. After the cure at 130 °C for 6 h and evaporation of the solvent at mild heating under vacuum (10 m Bar), the electrode disc (14 mm diameter) was an average mass coating of 2 mg·cm^−2^.

The lithium polymer cell (Li|LFP) was assembled by placing it in a proper order: a lithium metal disc as an anode, a film of the gelled electrolyte, and the LFP as a cathode in a coin cell (CR2032). The cell system was prepared in the Argon filled glove box.

## 3. Results & Discussion

### 3.1. Transport Properties in Phosphonium Electrolyte

#### 3.1.1. Ionic Conductivity

**Figure 1:**
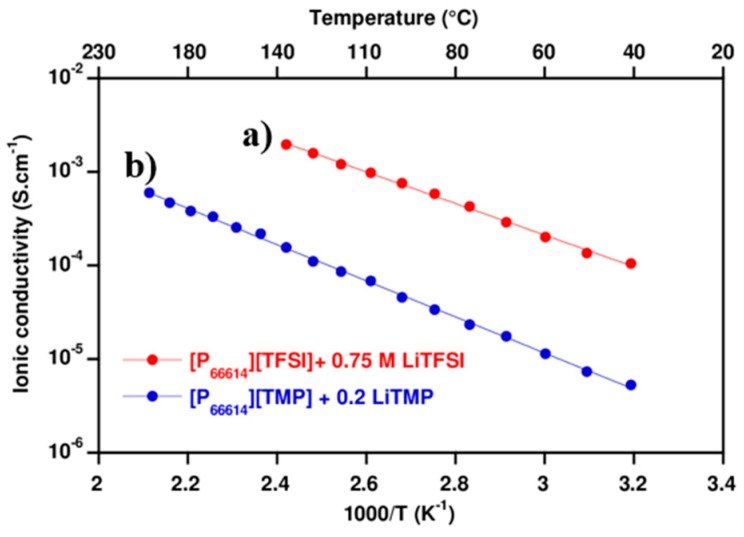
Ionic conductivity of phosphonium electrolytes with two types of anion: (a) [P_66614_][TFSI] with 0.75 M of LiTFSI and (b) [P_66614_][TMP] with 0.2 M of LiTMP.

The amount of lithium salt corresponding to the maximum soluble in ionic liquid was determined to 0.75 M for LiTFSI in [P_66614_][TFSI] and 0.2 M for LiTMP in [P_66614_][TMP]. The ionic conductivity of phosphonium electrolytes measured by Electrochemical Impedance Spectroscopy (EIS) and calculated by Equation (1) is reported in Figure 1.

(1) $$\sigma =\;\frac{d}{S.{\;R}_{\mathrm{el}}}$$

where d is the electrolyte thickness (cm), S the activity area (cm^2^), and Rel the resistance of the electrolyte (Ω).

The ionic conductivity of electrolyte [TFSI] is two decades higher compared to electrolyte [TMP] one. 1. Figure 1 shows that the log of the measured ionic conductivity is linear with respect to 1/T (the Arrhenius law). By modelling with an Arrhenius law, the activation energy could be determined and associated to the ion mobility in the electrolyte. The values obtained are summarized in Table 2. The activation energy of electrolyte [TFSI] (470 J·mol^−1^) is lower than electrolyte [TMP] one (530 J·mol^−1^), which can explain a better ion mobility in the electrolyte [TFSI]. In addition, this difference of ionic conductivity between [TFSI] and [TMP] based electrolytes can be explained by a higher salt concentration for electrolyte [TFSI] due to the difference in solubility. Moreover, TMP is a larger and non-fluorinated anion that has a significant influence on the mobility of the global ion in the electrolyte. The dissociation coefficient of the LiTMP is largely lower than for LiTFSI, due to the lower acidity of the corresponding acid.

#### 3.1.2. Diffusion Coefficient

The ionic conductivity of the electrolyte corresponds to the ion mobility present in the electrolyte. To understand the mobility of ions in the electrolyte, the diffusion coefficients of proton (D[H]) and lithium (D[Li]) were investigated by Pulsed Field Gradient NMR and are presented in Table 3.

For the electrolyte [TFSI], the measurement of D[H] associated to the phosphonium cation is of 9.25 × 10^−8^ cm^2^·s^−1^. This result is in agreement with the literature [29]. MacFarlan and al. have measured two diffusion coefficients corresponding to anion and cation. The values obtained for the cation is the same order (10^−8^ cm^2^·s^−1^). The diffusion coefficient D[Li] (10^−9^ cm^2^ s^−1^) is lower than the diffusion coefficient D[H]. The lithium ion has a lower mobility in the IL [P_66614_][TFSI]. The structuration of the electrolyte and particularly the complex steric hindrance around the lithium ions decreases its mobility compared to the mobility of the phosphonium cation. An increase of temperature would allow for getting a higher lithium mobility.

For the electrolyte [TMP], the value of D[H] is the same order as the electrolyte [TFSI] one. But, no signal is observed for the lithium ion. Two main assumptions can be made to explain this phenomenon: (i) a low dissociation of lithium salt in the electrolyte or (ii) a poor lithium salt solubility in the IL [P_66614_][TMP].

#### 3.1.3. Lithium Solubility

**Figure 2:**
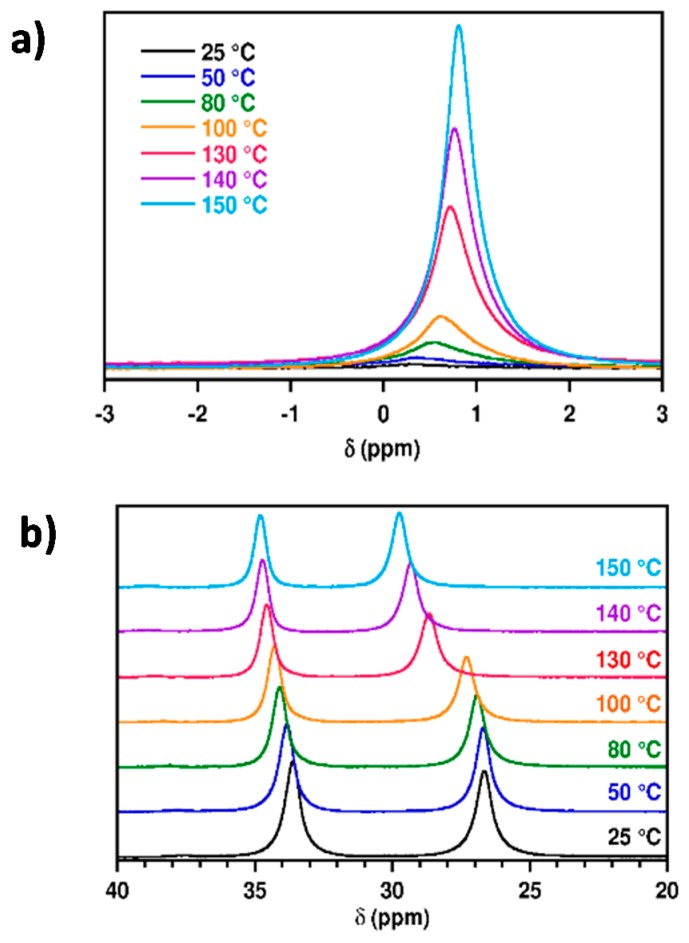
^7^Li NMR (**a**) and ^31^P NMR (**b**) of electrolyte [TMP] ([P_66614_][TMP] + 0.2 M LiTMP) at different temperatures.

To understand the absence of lithium signal, ^7^Li and ^31^P NMR spectroscopy were carried out under high-resolution liquid-state NMR conditions, in a temperature range from 25 °C up to 150 °C. The recorded spectra are shown in Figure 2.

For the spectra of ^7^Li NMR (Figure 2a), only one peak was observed, corresponding to a lithiated species. However, the chemical shifts and the peaks widths clearly change with the temperature; the linewidth becomes larger as the temperature decreases. These changes can be unambiguously assigned to the solubility feature of the lithiated species in the medium. In fact, the temperature increases the solubility of salt in the solvent. It means that between 100 °C and 25 °C the salt becomes less and less mobile due to its lower solubility. Around 25 °C, the NMR signal completely disappears under liquid-state NMR conditions. As a matter of fact, a broad line cannot be observed using the high-resolution liquid-state NMR probe. However, using solid-state NMR conditions, a ^7^Li signal of 200 ppm (not shown in this article) could be recorded, which is fully consistent with the immobilized state of the salt. The line broadening is due to a chemical shift in the anisotropy and dipolar interactions. This observation is fully consistent with the fact that no signal was measured during the analysis of diffusion coefficient at 54 °C and 86 °C. The signal of lithium becomes finer and more intense from 130 °C.

The ^31^P NMR spectrum determines the temperature from which the lithium salt is soluble through the phosphinate anion, which is common to lithium salt and to IL. The peak associated to the phosphonium cation (34 ppm at 25 °C) was used as a reference since it appeared unchanged regardless of the salt solubility. The second peak at 27 ppm at 25 °C corresponds at the anion phosphinate signal [30]. As for the ^7^Li NMR, an evolution of the chemical shift of the two ^31^P signals with the temperature can be observed. The cation peak shifts about 1.2 ppm, while a larger shift of about 3.1 ppm is observed for the anion. The temperature dependence of the chemical shift of the anion clearly presents two regimes since a significant change appears between 100 °C and 130 °C. That could be assigned to a chemical environment change of the anion associated to the better solubility of the lithium salt above 100 °C.

**Figure 3:**
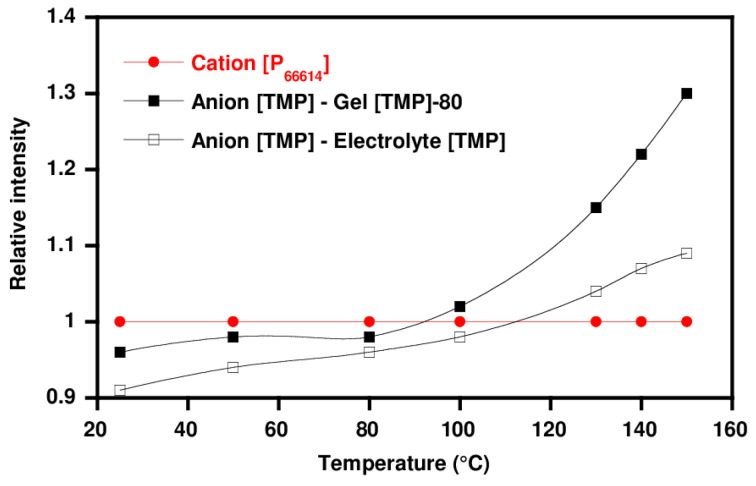
Evolution of the normalized relative intensity of the phosphinate anion compared to the relative intensity of the cation as a function of the temperature for the electrolyte [TMP] ([P_66614_][TMP] + 0.2 M LiTMP).

The relative intensity of the anion peak compared to of the relative intensity of the cation peak (considered as reference) as a function of temperature is presented in Figure 3. The relative intensity of the anion peak is higher than 1 from 100 °C, which indicates the beginning of the lithium salt solubilization in the medium. For an optimal solubilization, it was necessary to use an operating temperature around 150 °C. This is a relatively high temperature for using an electrolyte in a lithium battery.

To conclude this part, the electrolyte [TFSI] presents a better ability to be used as an electrolyte in a lithium battery. Therefore, this gelled electrolyte [TFSI] was chosen and its properties were investigated. Specifically, the electrochemical performance in the Li|LFP cell was discussed.

### 3.2. IL Confinement within the Epoxy Network

#### 3.2.1. Effect of IL Content on the Exudation

Table 4 presents the exudation behavior of cured epoxy networks modified with varying electrolyte content. The electrolyte exhibits a high miscibility since a maximum of ionic liquid of 70 wt % can be incorporated in a gel state. However, when more electrolyte is added, the sample becomes difficult to handle.

#### 3.2.2. Effect of IL on Reaction Progress

A NIR analysis was investigated to understand the differences in the reaction mechanism within an epoxy prepolymer-amine system with and without ionic liquids. In all the cases, the curing procedure was kept the same; i.e., 6 h at 110 °C and 6 h at 120 °C. The different band assignments are listed in Table 5 [31]. At the beginning, the spectrum of the initial mixture displays an absorption peak of the epoxy group at 4530 cm^−1^, an absorption peak of the primary amine group at 4935 cm^−1^, and an absorption peak of secondary and primary amine groups at 6500 cm^−1^. The evolution of these peaks follows the reaction, particularly after the gel time.

The cross-linking process is quantified from the decrease of these absorption peaks at 4530 and 4935 cm^−1^. The conversion (α) of epoxy group is calculated from the relationship (2) [31].

(2) $$\alpha_{\mathrm{epoxy},t}=1-\frac{{\left(A_{\mathrm{epoxy},4530}\right)}_{t}}{{\left(A_{\mathrm{epoxy},4530}\right)}_{t=0}}$$

where A_epoxy_ are the areas of the epoxy peak at the curing time (t) and at the start of the reaction (t = 0).

Equation (3) can also be applied to calculate the conversion of the primary amine characterized by the band at 4935 cm^−1^:

(3) $$\alpha_{\mathrm{PA},t}=1-\frac{{\left(A_{\mathrm{PA},4935}\right)}_{t}}{{\left(A_{\mathrm{PA},4935}\right)}_{t=0}}$$

where PA represents the primary amine (PA) absorption band at 4935 cm^−1^.

Equation (4) permits to follow the evolution of the both primary and secondary amine functions (A) at 6500 cm^−1^:

(4) $$\alpha_{A,t}=1-\frac{{\left(A_{A,6500}\right)}_{t}}{{\left(A_{A,6500}\right)}_{t=0}}$$

where A represents the primary and secondary amine (A) absorption band at 6500 cm^−1^. The evolution of secondary amine can be determined with Equation (5):

(5) $$\beta_{\mathrm{SA},t}={\;\alpha }_{\mathrm{PA},t}-{\;\alpha }_{A,t}$$

**Figure 4:**
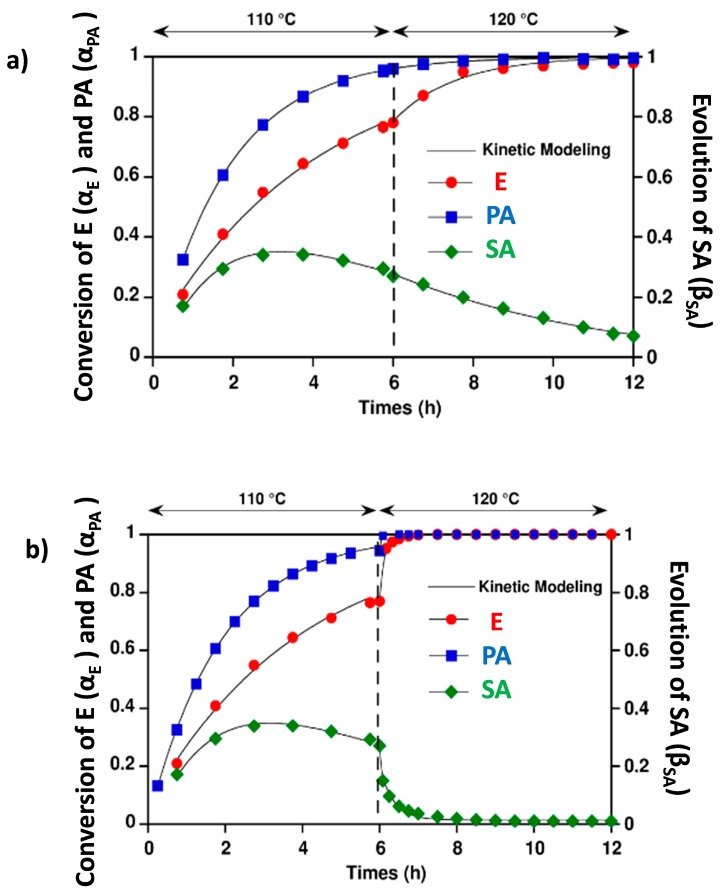
Epoxy conversion (E), and primary amine (PA) and evolution of secondary amine (SA) during the reaction time at 110 °C for 6 h and at 120 °C for 6 h of (**a**) neat PPO-Jeffamine networks compared to (**b**) PPO-Jeffamine/Electrolyte system with 50 wt % of ([P_66614_][TFSI] + 0.75 M LiTFSI).

Thus the conversion of the epoxide groups (E), primary amine (PA), and the evolution of secondary amine versus reaction time were plotted in Figure 4 for 50 wt % of ([P_66614_][TFSI + 0.75 M LiTFSI). The kinetic modelling of epoxy and primary amine functions conversion is made according to a first-order kinetic law according to Equation (6):

(6) $$\frac{\mathrm{dx}}{\mathrm{dt}}=k\left(1-x\right)x=x_{\infty }-x_{0}\exp^{-\mathrm{kt}}$$

where x is the conversion, k is the kinetic constant of the reaction, x_∞_ is the conversion at an infinite time (usually equal to 1), x_0_ is the conversion at t = 0 or t = 6 h. The kinetic constants from the modelling are shown in Table 6.

For the neat PPO-Jeffamine network, during the first step of curing, the reaction between the epoxy and primary amine groups is favored since the kinetic constant associated to the primary amine is higher than the one corresponding to the secondary amine (kAP = 0.534 h^−1/2^). The temperature increase results in the kinetic constants of the epoxy and secondary amines function higher. The function conversion does not reach 100% along the curing process because the temperature is limited at 120 °C during the second curing cycle.

The addition of the 50 wt % electrolyte induces a strong increase of kinetic constants of epoxy, as well as the secondary and primary amine functions. A total conversion of the amine and epoxy functions is reached at the end of the curing process. Therefore, the addition of electrolyte has a catalytic effect on the polymerization of the PPO-Jeffamine network. These results are in agreement with literature [32,33] that proves lithium salt LiTFSI, which is a lewis acid, helps the opening of the epoxy ring and thus catalyzes the polymerization of the epoxy amine network. This effect is different compared to our previous works where the anions of ionic liquids initiated the epoxy etherification [27].

In summary, it was shown that the ionic liquid [P_66614_][TFSI] presents a good compatibility with the network. The NIR analysis for monitoring the polymerization kinetics highlights the effect of the anion as a catalyzer of PPO-Jeffamine polymerization. Let us now consider the influence of the electrolyte on the final properties of the epoxy networks.

### 3.3. Influence of the Electrolyte on the Final Properties of Epoxy Based-Networks

#### 3.3.1. On the Thermal Stability of Networks

**Figure 5:**
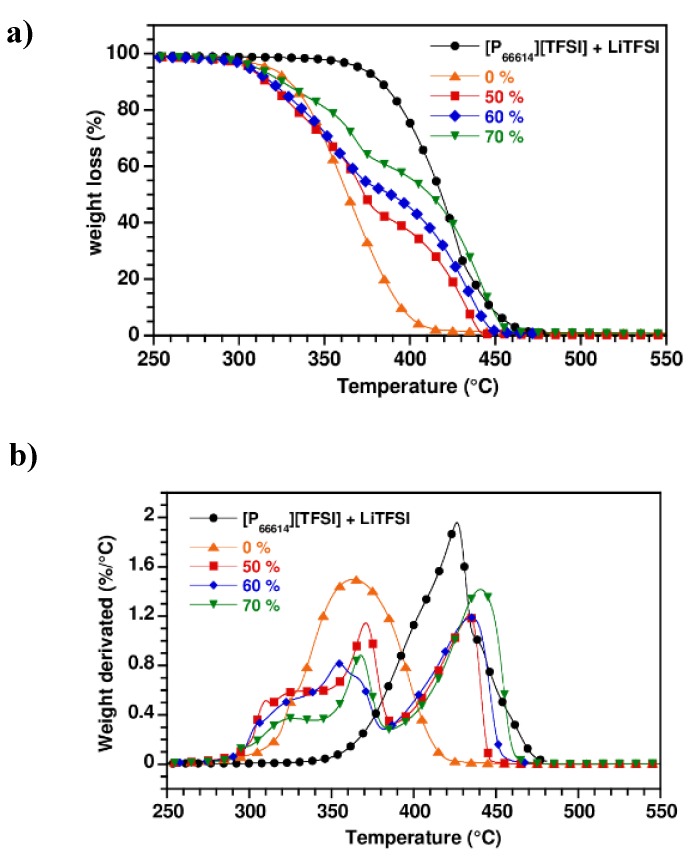
Thermo-gravimetric analyses (TGA) (**a**) and dynamical mechanical analysis (DTG) (**b**) of electrolyte ([P_66614_][TFSI] + 0.75 M LiTFSI) alone and introduced in PPO-Jeffamine networks prepared with an electrolyte varying content (heating ramp: 10 K·min^−1^; atmosphere: Nitrogen).

The thermal stability of PPO-Jeffamine/Electrolyte networks was investigated by thermogravimetric analysis (TGA) and compared to a neat epoxy-amine network and pure electrolyte. Figure 5 gives the weight loss and the corresponding derivative (DTG) of the networks as a function of the electrolyte content.

In Figure 5, the thermal stability of the electrolyte (Td5% = 370 °C) is much higher than the epoxy network (Td5% = 319 °C). The gelled electrolytes have an intermediate thermal stability between one of the host networks and the electrolyte one, depending on the electrolyte content. The DTG (Figure 5b) clearly identifies two degradation peaks corresponding to the degradation of the network between 300 and 385 °C and the second one to the degradation of the electrolyte localized between 385 and 460 °C. All the gelled samples present a high thermal stability but are limited by the epoxy network (Td5% = 310 °C).

#### 3.3.2. On the Viscoelastic Properties

The effect of electrolyte content on the viscoelastic properties of PPO-Jeffamine networks was investigated by dynamical mechanical analysis. The relaxation temperature (Tα), defined as the maximum value of tan δ and the storage modulus E′ at rubbery state at −10 °C are summarized in Table 7.

In both cases, one single peak of relaxation was observed, suggesting a homogeneous network without a macroscale phase separation phenomenon. Then, the addition of the electrolyte induces systematically a slight increase of Tα from −49 °C to −43.0 °C in the presence of 70 wt % of electrolyte. On the other side, the addition of the electrolyte leads to a strong decrease of storage modulus E′ at a rubbery state, which can be attributed at a decrease of network crosslink density [34,35]. Thus, E′ decreases 80% in the presence of 70 wt % of electrolyte. So, the addition of a large amount of electrolyte in the gel significantly impacts the mechanical strength of the gelled electrolyte by increasing the gel flexibility and making it even more difficult to handle. This observation can be correlated at the diluted effect of the medium, where the probability of the prepolymers to meet each other decreases with the addition of the electrolyte. The semi-diluted medium then promotes intra-molecular reactions like cyclization are considered defective and increase the apparent elasticity of the network.

### 3.4. Effect of Electrolyte on the Transport and Electrochemical Properties

#### 3.4.1. Transport Properties

To confirm the potential use of this system as a gelled electrolyte for lithium-ion battery, a film with low thickness (<100 µm) was prepared by solvent casting. The gelled electrolytes were prepared with different amounts of electrolyte containing [P_66614_][TFSI]/lithium salt (LiTFSI). The ionic conductivities were measured by Electrochemical Impedance Spectroscopy (EIS) and calculated by Equation (1).

**Figure 6:**
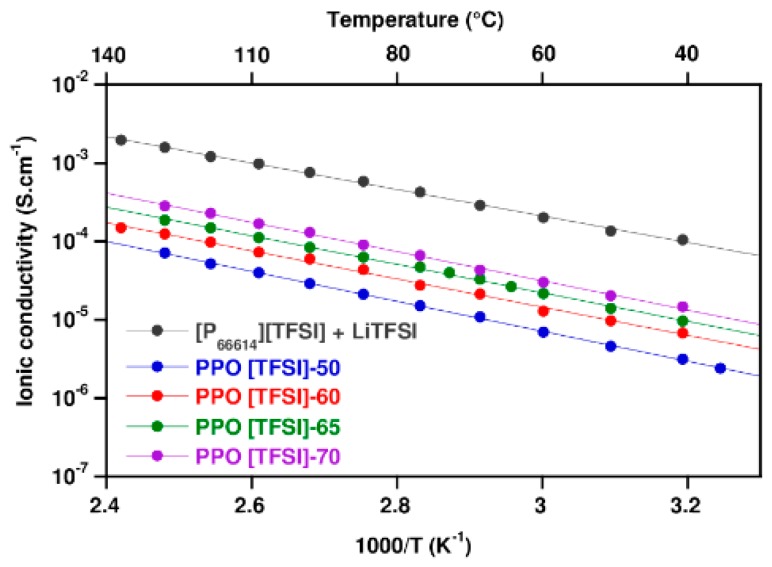
Ionic conductivity of gelled electrolyte with different amount of electrolyte ([P_66614_][TFSI] with 0.75 M of LiTFSI).

Figure 6 shows the temperature dependence (in the range of 20–140 °C) of the ionic conductivities of the gelled electrolyte with different amounts of electrolyte in comparison with the neat electrolyte. The ionic conductivity of the gelled electrolyte increases both with the amount of electrolyte and with the temperature with a maximum value of 0.13 mS·cm^−1^ at 100 °C for 70 wt % electrolyte based gel. This phenomenon results from a higher number of ions and faster ion movements since it is thermally activated and leads to higher ionic conductivity. The ionic conductivity of the gelled electrolyte is lower than the neat electrolyte, because of the decrease of ion mobility confined in the host network.

By modelling the ionic conductivity plots with an Arrhenius law, the activation energy values were determined and reported in Table 8. These Ea values are independent on the electrolyte content because only the electrolyte trapped between the polymer chains is responsible for the ionic conduction.

#### 3.4.2. Electrochemical Stability

**Figure 7:**
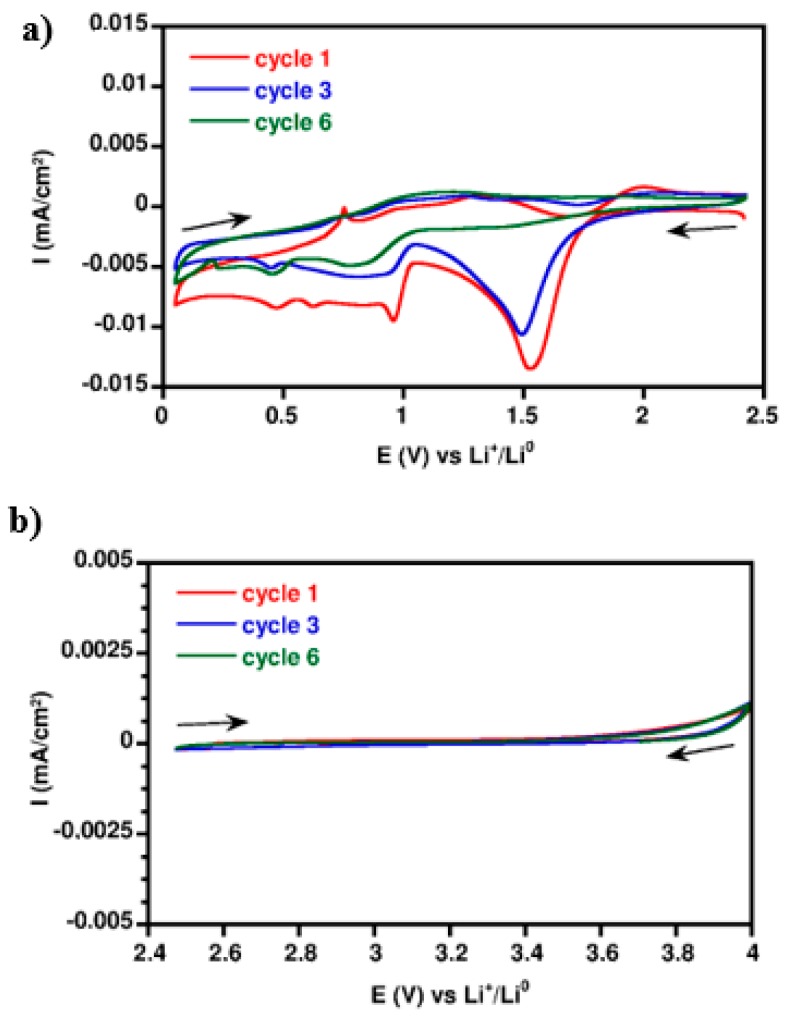
Cyclic voltammetry of gelled electrolyte prepared with 70 wt % of electrolyte (**a**) in reduction to 0.05 V vs. Li^+^/Li^0^ and (**b**) in oxidation to 4 V vs. Li^+^/Li^0.^

To confirm the electrochemical stability of the gelled electrolyte, a cyclic voltammetry (CV) has been used to know this electrochemical stability window. The CV curves are shown in Figure 7. The measurement has been performed in coin cells. The working electrode is a stainless steel disk. The counter electrode is metallic lithium, acting as well as a reference electrode.

In the reduction (Figure 7a), the electrochemical phenomenon at 1.5 V and 1 V are observed with a current intensity measured at −0.014 mA·cm^−2^ and at 0.01 mA·cm^−2^, respectively, for the first cycle. This phenomenon, observed as well for the electrolyte [TFSI], was attributed to the formation of a passivation layer that stabilizes at the 6th cycle. As the electrolyte is based on ionic liquids, the SEI should be different, as in conventional carbonate based electrolytes. The nature of the passivation layer is attributed to the chemical nature of the IL. In oxidation (Figure 7b), the current measured is very low (<2 µA·cm^−2^). Thus, it is possible to consider the gelled electrolyte is electrochemically stable with stainless steel to 4 V vs. Li+/Li^0^.

The cathodic and anodic limiting potentials are about 0.05 V and 4 V vs. Li+/Li^0^, respectively, and ESW is 3.95. These results suggest that gelled electrolyte PPO [TFSI]−70 is a good candidate to be used as an electrolyte in lithium ion batteries with the metallic lithium anode and the LiFePO_4_, with an active plateau at about 3.4 V vs. Li^+^/Li^0.3^.

#### 3.4.3. Cycling Performance of Li|LiFePO_4_ Cell

**Figure 8:**
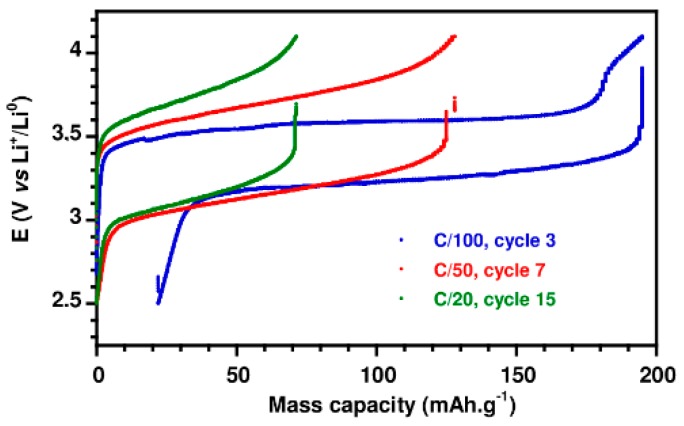
Cycling performances of Li|LiFePO_4_ cell at different current densities (C/100, C/50 and C/20) between 2.5 V and 4 V at 100 °C of gelled electrolyte prepared with 70 wt % of electrolyte.

The gelled electrolyte was tested in the Li|LiFePO_4_ cell at different current densities (C/100, C/50, and C/20) between 2.5 V and 4 V at 100 °C. The results are presented for the cycle 3, 7 and 15 in Figure 8.

The charge/discharge curves for the cell under a low current rate (C/100) are relatively flat lines at about 3.3 and 3.5 V, respectively. The curves obtained for the higher current rates (C/50 and C/20) deviate from this linear behavior. This can be explained by a lower ion diffusion within the electrolyte and at the electrode/electrolyte interface.

**Figure 9:**
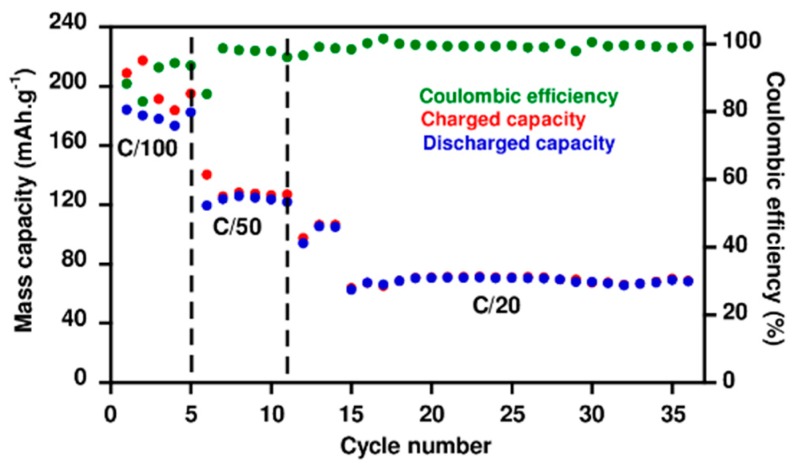
Cycling performance of the Li|PPO [TFSI]−70|LiFePO_4_ cell at indicating rates at 100 °C.

The Li|PPO [TFSI]−70|LiFePO_4_ shows a good cycling performance, with large charge/discharge capacities ranging from 210 m Ah·g^−1^ (slightly upper of the theoretical capacity) at C/100 and the initial coulombic efficiency is ~90% (Figure 9). The additional current consumption in charge was attributed to the degradation of the gelled electrolyte. When the current rate was increased to C/50, and then to C/20, the charge and discharge capacity was approximately the same with a better coulombic efficiency. However, the specific capacity was decreased to around 120 m Ah·g^−1^ then to 70 m Ah·g^−1^, which corresponds to 40% of the theoretical capacity. This decrease could be correlated to the lower ionic conductivity of the gelled electrolyte, which could induce an additional ohmic resistance and concentration gradients within the electrolyte. Therefore, the charge transfer could be more difficult to the electrodes and the polarization of the cell would be increased. Nevertheless, a good cycle stability was observed at C/20 over 20 cycles with a capacity of around 70 m Ah·g^−1^.

## 4. Conclusions

In this work, the transport properties of different phosphonium electrolytes denoted [P_66614_][TMP] + LiTMP and [P_66614_][TFSI] LiTFSI were investigated. We have shown that the electrolyte [TMP] have a low solubility of the lithium salt at ambient temperature. The NMR analysis determines the operating temperature at 150 °C. This temperature is too high to consider its use as an electrolyte for lithium battery. However, the electrolyte [TFSI] has shown a better ability for this use.

So, the electrolyte [TFSI] was confined within epoxy-amine networks. The influence of the electrolyte on the polymerization kinetics as well as on the final properties (thermal and mechanical) of the epoxy networks was performed. Thus, we have demonstrated that the electrolyte [TFSI] catalyzed the polymerization of the epoxy/amine prepolymer. We have highlighted the ability to develop flexible epoxy networks swollen with high amounts of electrolyte [TFSI] (70 wt %).

The gelled electrolyte has an ionic conductivity of 0.13 mS·cm^−1^ at 100 °C with an electrochemical stability window at 3.95 V. The test of cycling performance has shown for the first time the possibility to use a phosphonium gelled electrolyte in the Li|LFP cell. A low current rate was necessary to obtain a theoretical capacity of the material. Thus, a proof of concept has been achieved. In summary, this system could find some applications in extreme conditions, as very high temperature use requires high safety protocols. For conventional applications, a modification of the system (shorter IL, different polymer) is required to fit the expected performances as well as an optimization of the cell and the understanding that some phenomena involved at the interface of the electrolyte/electrode require further study.

## Figures and Tables

**Table 1:** Chemical structures and characteristics of the main compounds.

Name	Structure	Characteristics
PPO	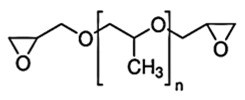	Sigma Aldrich; 320 g/eq
Jeffamine^®^D2000	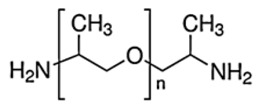	Supplied by Huntsmann; 514 g/eq
[P_66614_][TMP]	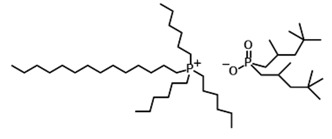	Supplied by Cytec; Molar mass = 773.27 g·mol^−1^ m.p. =−72 °C Td max = 350 °C
[P_66614_][TFSI]	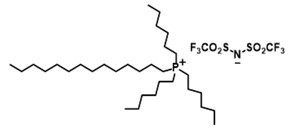	Supplied by Cytec; Molar mass = 764.0 g·mol^−1^ m.p. = −72.4 °C Td = 450 °C
LiTFSI	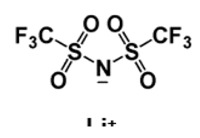	Supplied by Sigma Aldrich; Molar mass = 287.0 g·mol^−1^
LiTMP	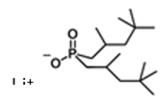	Supplied by Solvionic Molar mass = 780.27 g·mol^−1^

**Table 2:** Activation energy and correlation coefficients of phosphonium electrolytes.

Sample	Ea (J·mol^−1^)	R^2^
[P_66614_][TFSI] + LiTFSI	470	0.999
[P_66614_][TMP] + LiTMP	530	0.998

**Table 3:** Diffusion coefficient (in cm^2^·s^−1^) of phosphonium electrolytes.

	T (°C)	[P_66614_][TFSI] + LiTFSI	T (°C)	[P_66614_][TMP] + LiTMP
D [H]	28.5	9.25 × 10^−8^	54	2.3 × 10^−8^
			86	1.1 × 10^−7^
D [Li]	21	9.35 × 10^−9^	54	No signal
			86	No signal

**Table 4:** Name and composition of different gelled electrolytes.

Sample	Composition (wt %)			Exudation
	Polymer	IL	LiTFSI	
PPO [TFSI]-50	50	41.0	9.0	No
PPO [TFSI]-60	40	49.2	10.8	No
PPO [TFSI]-65	35	53.3	11.7	No
PPO [TFSI]-70	30	57.4	12.6	No

**Table 5:** Near infrared (IR) Band Assignments of PPO-Jeffamine polymerization.

Wavenumber (cm^−1^)	Peak Assignment
7099	O–H overtone
6600–6480	Primary and Secondary amine combination band Overtones of N–H stretching
6072	Terminal epoxy first overtone of C–H stretching
5880–5500	C–H overtone (CH_2_, CH_3_)
5249	−OH due to moisture (O–H asymmetric stretching and bending)
4935	Primary amine combination band N–H stretching and bending
4530	Epoxy combination band (C–H stretching and epoxy ring breathing)

**Table 6:** The kinetic constants (h^−1/2^) of reaction for epoxy functions (k_E_), for primary amine functions (k_PA_) and for secondary amine functions (k_SA_) and correlation coefficients.

System	PPO-Jeffamine		PPO-Jeffamine + ([P_66614_][TFSI] + LiTFSI)	
Step of Curing	First Step	Second Step	First Step	Second Step
k_E_ (h^−1/2^)	0.272	0.988	0.268	7.989
(R^2^)	(0.994)	(0.996)	(0.989)	(0.993)
k_PA_ (h^−1/2^)	0.534	0.534	0.532	30.266
(R^2^)	(0.999)	(0.999)	(0.999)	(0.999)
k_SA_ (h^−1/2^)	0.190	0.243	0.186	3.039
(R^2^)	(0.988)	(0.996)	(0.990)	(0.990)

**Table 7:** DMA data of PPO-Jeffamine/Electrolyte networks.

Electrolyte Content	Tα (°C)	Rubbery State E′ (MPa)
(wt %)		
0	−49.2	0.33
50	−48.8	0.25
60	−46.9	0.20
70	−43.0	0.06

**Table 8:** Activation energy and correlation coefficients of gelled electrolyte.

Sample	Ea (J·mol^−1^)	R^2^
[P_66614_][TFSI] + LiTFSI	440	0.999
PPO [TFSI]-50	500	0.999
PPO [TFSI]-60	470	0.997
PPO [TFSI]-65	475	0.999
PPO [TFSI]-70	490	0.999
